# IMPLEMENTATION OF COMMUNITY-BASED PALLIATIVE CARE: A SYSTEMATIC SCOPING REVIEW

**DOI:** 10.1093/geroni/igae098.0753

**Published:** 2024-12-31

**Authors:** Dorian Ho, Nicole Dissault, Judith Vick, Lesley Skalla, Haripriya Dukkipati, Jessica Ma, Brystana Kaufman

**Affiliations:** Duke-Margolis Institute for Health Policy, New Providence, New Jersey, United States; Duke University, Durham, North Carolina, United States; Yale University School of Medicine, New Haven, Connecticut, United States; Duke University, Durham, North Carolina, United States; Duke University, Durham, North Carolina, United States; Duke University School of Medicine, Durham, North Carolina, United States; Duke University School of Medicine, Durham, North Carolina, United States

## Abstract

Community-based palliative care (CBPC) models, which include palliative care (PC) delivered in outpatient clinics and homes, improve quality of life for people living with serious illness and their care partners. CBPC access has been expanding across the United States, with the goal of delivering PC earlier in the trajectory of serious illness care. This systematic scoping review aimed to summarize provider perspectives regarding barriers and facilitators to CBPC program implementation. MEDLINE and CINAHL were searched for peer-reviewed qualitative research published between 1/1/2010 and 1/9/2024 that included provider perspectives on CBPC, excluding hospice or end-of-life care. Two reviewers screened articles and extracted themes. Framework synthesis was used to categorize themes into provider, organization, and environmental levels according to the PRISM (Practical, Robust Implementation and Sustainability Model) framework. Thirty-five articles were included, varying by providers (PC providers, referring providers, administrators) and practice settings (PC clinic, specialty-embedded PC clinic, home). At the provider level, misperceptions of PC by referring providers, poor inter-provider communication, and unclear roles were perceived barriers to CBPC. Conversely, multidisciplinary PC teams, referring provider PC education, and collaboration were perceived facilitators. At the organizational level, time constraints were perceived barriers while leadership buy-in, shared medical records, and colocation of PC into specialty clinics were perceived facilitators. At the environment level, limited PC workforce and PC reimbursement were perceived barriers. Few articles addressed cultural sensitivity; standardized performance data; and social needs resources. Successful CBPC implementation requires addressing factors at the provider, organizational, and environmental levels.

